# SARS-Arena: Sequence and Structure-Guided Selection of Conserved Peptides from SARS-related Coronaviruses for Novel Vaccine Development

**DOI:** 10.3389/fimmu.2022.931155

**Published:** 2022-07-12

**Authors:** Mauricio Menegatti Rigo, Romanos Fasoulis, Anja Conev, Sarah Hall-Swan, Dinler Amaral Antunes, Lydia E. Kavraki

**Affiliations:** ^1^ Kavraki Lab, Department of Computer Science, Rice University, Houston, TX, United States; ^2^ Antunes Lab, Center for Nuclear Receptors and Cell Signaling, Department of Biology and Biochemistry, University of Houston, Houston, TX, United States

**Keywords:** SARS-CoV-2, SARS-CoV, protein sequence alignment, structural modeling, HLA-Arena, nucleocapsid protein, pHLA scoring

## Abstract

The pandemic caused by the SARS-CoV-2 virus, the agent responsible for the COVID-19 disease, has affected millions of people worldwide. There is constant search for new therapies to either prevent or mitigate the disease. Fortunately, we have observed the successful development of multiple vaccines. Most of them are focused on one viral envelope protein, the spike protein. However, such focused approaches may contribute for the rise of new variants, fueled by the constant selection pressure on envelope proteins, and the widespread dispersion of coronaviruses in nature. Therefore, it is important to examine other proteins, preferentially those that are less susceptible to selection pressure, such as the nucleocapsid (N) protein. Even though the N protein is less accessible to humoral response, peptides from its conserved regions can be presented by class I Human Leukocyte Antigen (HLA) molecules, eliciting an immune response mediated by T-cells. Given the increased number of protein sequences deposited in biological databases daily and the N protein conservation among viral strains, computational methods can be leveraged to discover potential new targets for SARS-CoV-2 and SARS-CoV-related viruses. Here we developed SARS-Arena, a user-friendly computational pipeline that can be used by practitioners of different levels of expertise for novel vaccine development. SARS-Arena combines sequence-based methods and structure-based analyses to (i) perform multiple sequence alignment (MSA) of SARS-CoV-related N protein sequences, (ii) recover candidate peptides of different lengths from conserved protein regions, and (iii) model the 3D structure of the conserved peptides in the context of different HLAs. We present two main Jupyter Notebook workflows that can help in the identification of new T-cell targets against SARS-CoV viruses. In fact, in a cross-reactive case study, our workflows identified a conserved N protein peptide (SPRWYFYYL) recognized by CD8^+^ T-cells in the context of HLA-B7^+^. SARS-Arena is available at https://github.com/KavrakiLab/SARS-Arena.

## 1 Introduction

In 2003, the Severe Acute Respiratory Syndrome Coronavirus (SARS-CoV) caused a pandemic that resulted in more than 8,096 cases and 774 deaths (1). This was not the first time coronaviruses cause epidemics in humans, and multiple strains of coronaviruses have been identified in bats and other organisms, serving as a warning about the risks for a new epidemic (2). Unfortunately, acknowledging the presence of circulating coronaviruses was not sufficient to avoid the current pandemic, caused by a novel strain of coronavirus called SARS-CoV-2 (3). SARS-CoV-2 is the etiologic agent responsible for the COVID-19 disease in humans. This new variant was identified at the end of 2019 and quickly spread to a pandemic level during the first months of 2020. The consequences of COVID-19 have been disastrous, both to individual health as well as to the economy (4). Massive vaccination campaigns across different countries have been crucial to helping the mitigation of COVID-19. However, the large reservoir of SARS-type viruses in the wild, and the well-known capacity of coronaviruses to undergo genetic recombination, highlights the continued risk for new pandemics in the future (2, 5, 6). Therefore, there is a need for effective vaccination strategies that would protect individuals against a broad range of SARS-like coronaviruses.

Because of the inverse correlation of protection between neutralizing antibodies and SARS-CoV-2 viral load (7, 8), envelope proteins - such as the spike (S) protein, have been used as the main target on currently approved human vaccines. However, envelope proteins are known to be more susceptible to selection pressure in comparison to inner viral proteins, and therefore more prone to mutations that can lead to resistance to treatment and decreased vaccine efficacy. During the SARS-CoV-2 pandemic, we did observe cases that led to an increase of infectiousness (e.g., D614G mutation) or transmissibility (e.g., B.1.1.7 variant, also called Alpha variant) (9, 10) driven mainly by mutations in envelope proteins. The variant B.1.617 (also called Delta variant), containing pivotal mutations on the S protein, rapidly became the dominant strain in several countries during 2021. The B.1.1.529 variant, named the Omicron variant, has more than 30 new mutations in the S protein and these mutations may contribute to improved infectiousness of SARS-CoV-2 (11). In other words, even with successful vaccines developed for SARS-CoV-2, it is unclear for how long the efficacy will persist. This is highlighted by a recent WHO statement on the need of updating current vaccines (https://www.who.int/news/).

Apart from the development of a strong humoral response (e.g., neutralizing antibodies), vaccination strategies also have to induce a protective, long-term, cell-mediated immunity (i.e., based on T-cell lymphocytes). Reports on SARS-related coronaviruses have shown that SARS-CoV-specific antibodies can significantly drop in the first 2 to 3 years after infection (12), while the SARS-CoV-specific T-cells can persist for more than a decade (13). T-cells recognize peptides displayed at the surface of infected cells by class I Human Leukocyte Antigens (HLAs). Therefore, peptide-based vaccines aiming at triggering T-cells can target any viral protein, and proteins with lower mutation rates in respect to envelope proteins would represent promising targets for broad-spectrum vaccine development (14).

One of these proteins is the nucleocapsid (N) protein. The N protein is a promising target for a multitude of reasons. Firstly, this protein is highly conserved even across different coronaviruses (15) and is highly immunogenic and expressed during the infection course (16). Moreover, it presents a low mutation rate compared to envelope proteins, mainly because this protein is not exposed on the surface of the virus and hence is less impacted by the antibody-mediated selective pressure (17). Additionally, studies have shown that SARS-recovered patients can present CD4^+^ and CD8^+^ T-cells that recognize multiple regions of the N protein; and long-lasting T-cell memory against N protein targets can persist for decades (18). Thus, the identification of peptide targets from the N protein can support the design of new vaccines and treatments focused on T-cell immune response.

The state-of-the-art for identifying peptide targets involves the use of computational methods to predict the binding of viral peptides to different HLA receptors. In this sense, sequence-based methods are widely used for this task (19–21). However, recent studies have highlighted that the accuracy and sensitivity of sequence-based methods vary widely across HLA alleles (22, 23). One way to improve accuracy/sensitivity would be including peptide-HLA (pHLA) structural features to complement sequence analysis. This was the basis that led to the development of HLA-Arena, a platform that combines sequence- and structure-based analysis of pHLA complexes (24). The addition of structural information from models obtained using HLA-Arena produced a higher rate of true positive and true negative HLA-binding predictions. HLA-Arena provided a proof-of-concept that both sequence-based and structure-based analyses can be combined into a single, user-friendly computational pipeline, complementing each other into a more reliable and more general consensus prediction. Since different datasets of SARS-CoV-2-peptides have already been identified using sequence-based methods for HLA binding prediction (25, 26), we expect that a combined approach using sequence and structural methods could be applied for the identification of peptide targets for novel vaccine development.

**Figure 1:**
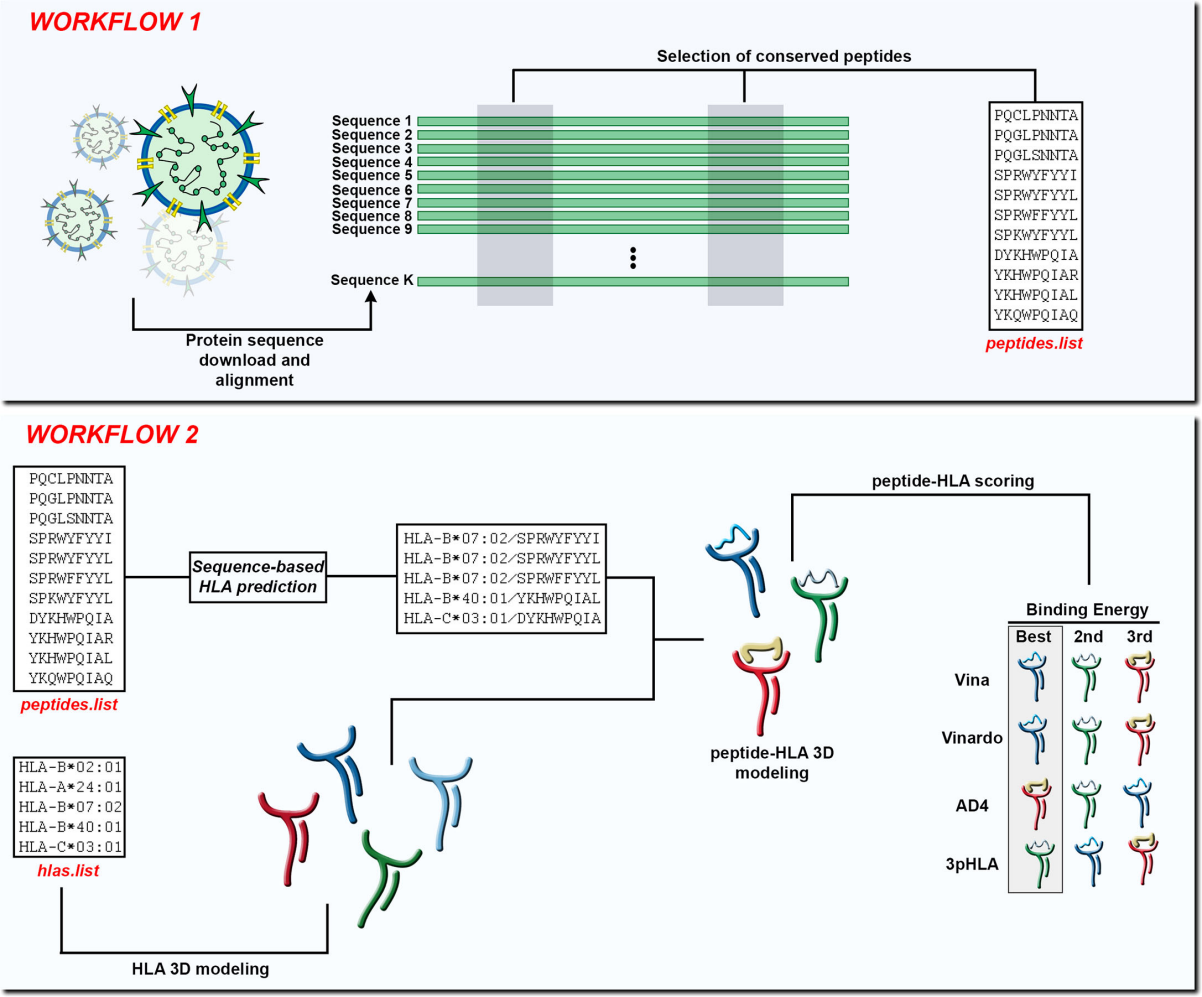
In Workflow 1, the input is a set of K protein sequences and the output is a list of peptides found in conserved regions (peptides.list). In Workflow 2, the input is the peptides.list from the previous workflow along with a list of HLA alleles (hlas.list) provided by the user. After using a sequence-based HLA binding affinity prediction tool, the peptides are modeled in the context of the chosen HLA molecules. At the end of Workflow 2 the peptide-HLA structures can be scored according to different scoring functions and the best choices can be presented to the user.

Here, we develop SARS-Arena, a pipeline comprised of two workflows that leverages the HLA-Arena environments. Using the first workflow (hereafter called Workflow 1) the user can perform multiple sequence alignment (MSA) of N protein sequences to identify and extract possible peptide targets from conserved regions. Using the second workflow (hereafter called Workflow 2) peptide targets are filtered based on a sequence-based HLA-binding prediction tool. Finally, following the structural modeling of the peptide-HLA complex, we apply a filtering step using well-known scoring functions for structural assessment (**Figure 1**). The output of Workflow 1 is a list of peptides found in conserved regions of N protein from SARS-CoV-2 or SARS-related coronaviruses. In Workflow 2, the output is the three-dimensional model of these peptides sorted according to different scoring functions in the context of different HLAs. We show the advantage of SARS-Arena through a case study to retrieve a well-known immunogenic N peptide and its variants from a set of protein sequences deposited at NCBI.

## 2 Materials and Methods

**Figure 2:**
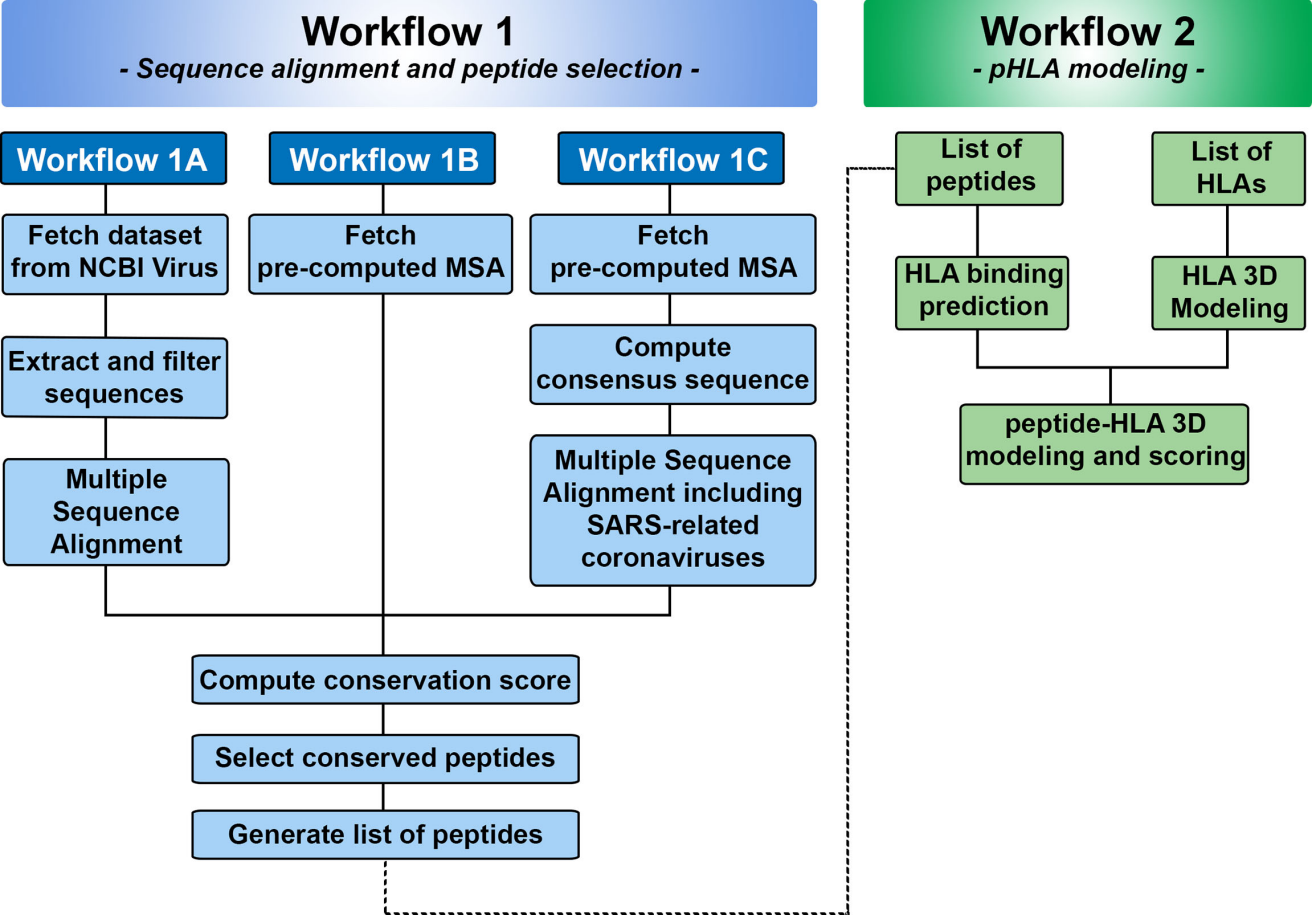
Overview of Workflows 1 and 2 in SARS-Arena. Workflow 1 is focused on sequence analysis and is organized in three parts: Workflow 1A, 1B, and 1C. Each workflow differs in the way the information is obtained for MSA. At the end of Workflow 1, a list of peptides is generated and can be used in the Workflow 2. Workflow 2 is focused on structural analysis and will return the 3D structure of each peptide in the context of a specific HLA.

We used Jupyter Notebook to create two workflows for SARS-Arena. The first workflow (Workflow 1) is designed to allow users to select conserved peptides from N protein MSA. Because the origin of the sequences and the alignment approach can differ, we subdivided Workflow 1 accordingly (see **Figure 2**). The second workflow (Workflow 2) is related to the modeling of conserved peptides in the context of different HLA molecules. To facilitate user experience we created a set of functions that can be accessed from the GitHub repository at https://github.com/KavrakiLab/SARS-Arena. SARS-Arena is also made available in a Docker image, which can be downloaded directly from Docker Hub (e.g., using the command line *docker pull kavrakilab/hla-arena:sars-arena*). The following subsections (2.1 to 2.5) will describe the methodologies we use in Workflow 1 and Workflow 2. Then Section 3 concentrates on the results we can obtain using the workflows.

### 2.1 SARS-CoV Sequences and Multiple Sequence Alignment

Workflow 1 can be used to select peptides contained in conserved regions of N protein sequences from SARS-CoV-2 (Workflow 1A and 1B) or SARS-related coronaviruses (Workflow 1C). In Workflow 1A, SARS-CoV-2 protein sequences are retrieved directly from NCBIVirus (27). In Workflow 1B and 1C, the N protein alignment is already pre-computed. In Workflow 1C, apart from the SARS-CoV-2 sequences, we also used a file with a total of 64 pre-defined N protein sequences from SARS-related coronaviruses. This file was created from information deposited at GenBank and can be augmented with more sequences depending on user needs. For the MSA, we used the MAFFT program (28) because of its capacity to parallelize jobs during sequence alignment. The time to finalize the alignment task depends on a set of variables, such as the number of sequences to be analyzed, the hardware available to run the alignment, and the number of available cores to be used for the parallel jobs. For this reason, we provide pre-computed MSAs in Workflow 1B and 1C. These MSAs were performed at the NOTS cluster (CRC Rice University) and stored at the Owl Research Infrastructure Open Nebula (ORION) Virtual Machines. The alignments are updated every week so that the users can work with the latest sequences released from NCBI.

### 2.2 Conservation Threshold

**Figure 3:**
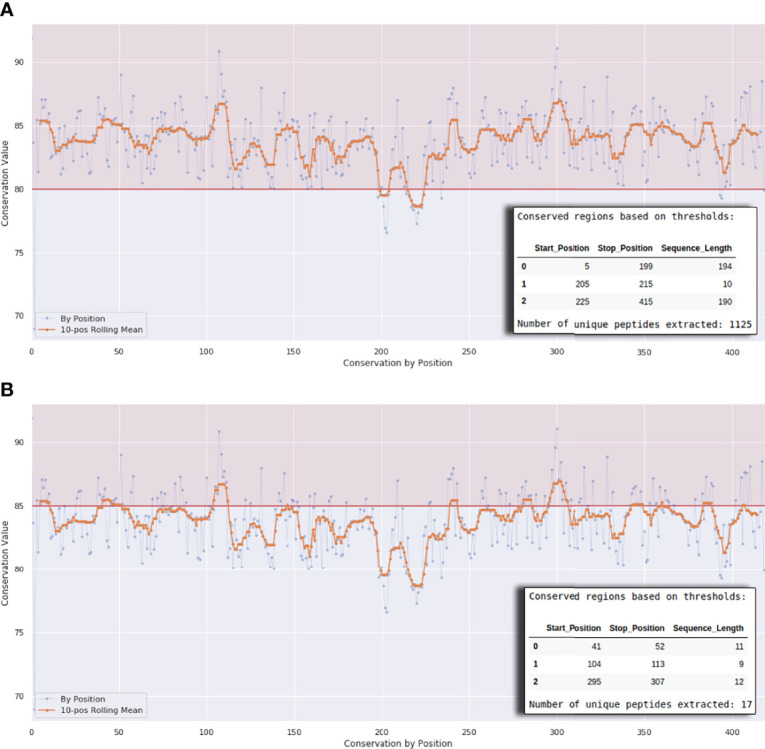
Interactive plot interface at the end of Workflows 1A, 1B, and 1C. The horizontal line in **(A)** defines a conservation threshold of 80% and in **(B)** a threshold of 85%. The x-axis represents the protein length. The number of peptides will vary according to this threshold, as exemplified by the small tables on the lower right corner of the graph.

In Workflow 1 the user needs to define a scoring method and a scoring matrix so that the level of conservation in the different parts of the aligned K protein sequences is calculated. We provide four different scoring methods - Jensen-Shannon divergence score (29), Shannon entropy (30), Property entropy (31), and Von Neumann entropy (32). We choose these scoring methods based on a previous publication by Capra and Singh (33). For scoring matrices, we used the well-known BLOSUM matrices (35, 40, 45, 50, 62, 80, and 100). The conservation cutoff can be modified using a interactive plot at the end of the workflow (**Figure 3**). As conservation values are different and not homogeneous for each position, we provide a Rolling Median Window Length cutoff variable. Alternatively, the user can set the cutoff variable value to 1 to take conservation as it is.

### 2.3 Sequence-Based HLA Binding Prediction

It is known that peptide-HLA binding is mainly driven by sequence features. For this reason, we used MHCflurry 2.0 (34) to perform a sequence-based HLA binding prediction. We used default parameters to run MHCflurry with a threshold set to 500nM. This value was chosen to recover strong and intermediate HLA peptide binders (35). Before proceeding, the user can use a interactive plot to modify the 500 nM threshold according to their needs.

### 2.4 HLA and Peptide-HLA Modeling

The sequence to structure conversion occurs in two phases. First, the user needs to input the HLAs of interest in order to pair the peptides obtained from Workflow 1. The name of the HLAs should be contained in a file called *hlas.list* file. We pull the sequences of the user-selected HLA alleles from the EBI database (36). After that, we use Modeller (37) to transform the sequence into a three-dimensional structure. We use a homology modeling approach and functions retrieved from HLA-Arena (24). Since HLAs are highly conserved molecules in terms of sequence and structure, we expect a high accuracy on the generated models. After that, we use APE-Gen (38) to build the peptide-HLA complex. APE-Gen is a tool that models peptide-HLA complexes using an iterative modeling approach where each iteration contains three key steps. First, the peptide backbone is anchored to HLA pockets (anchor positions) using backbone termini templates. Next, the backbone is completed using a Random Coordinate Descent loop modeling tool (39) to generate a set of possible backbone conformations. Finally, side chains are added and a local optimization is performed to correct steric clashes. APE-Gen generates an ensemble of conformations, which are all stored for further analysis if desired. By default, however, only the pHLA structure with the lowest energy (i.e., best binding) is selected for subsequent analysis.

### 2.5 Scoring Functions

Binding energy of modeled peptide-HLA structures can be evaluated within Workflow 2 using four integrated scoring functions: AutoDock4 (40), Vina (41), Vinardo (42) and 3pHLA-score (43). AutoDock4 score is based on an empirical free energy forcefield and is a part of a widely used protein-ligand docking tool. Vina and Vinardo scores are empirical scoring functions. They both originate from the Vina docking tool. The 3pHLA-score is a recently developed scoring function tailored for pHLA structures produced by APE-Gen and based on Rosetta’s ref2015 score (44). It uses a novel per-peptide-position training approach and consists of per-allele trained modules. It currently supports 28 HLA alleles. The binding energies estimated with the proposed functions are then used to rank the peptides and further refine the list of selected targets. An interactive plot is provided to visualize the scores and allow for dynamic thresholding.

## 3 Results

We created two independent workflows that automatically retrieve peptides located in conserved regions of N protein from SARS-CoV-2 and SARS-related coronaviruses (Workflow 1), and model the three-dimensional structure of these peptides in the context of specific HLAs (Workflow 2) (**Figure 2**). The results we obtain from each workflow are explained in the next sessions.

### 3.1 Workflow 1: Sequence Alignment and Peptide Selection

The first part of SARS-Arena is the sequence alignment and peptide selection, which is coded inside Workflow 1. As described above, we organized this first workflow into three independent parts: Workflow 1A, Workflow 1B, and Workflow 1C. The output of each workflow is the same: a list of peptides obtained from conserved protein regions (**Figure 2**). The different workflows were created to accomodate different inputs.

#### 3.1.1 Workflow 1A

Workflow 1A allows users to run the MSA of SARS-CoV-2 proteins *in loco*. The maximum limit of sequences to be analyzed will depend on the user’s system hardware. For this reason, we recommend using this workflow when the number of total protein sequences to be analyzed is small (approximately 50,000 sequences). Workflow 1A consists of the following steps. As a preliminary step, the necessary python packages are imported, and the working directories where intermediate files are sorted are also defined. After that, the protein sequences from NCBI Virus are extracted based on a set of parameters. These parameters include choosing (i) the virus strain, (ii) the protein (N protein is the default option), (iii) the completeness of genomes, (iv) the host, (v) the use of only reference sequences or all sequences available, (vi) the geographic region, (vii) the isolation source, (viii) the Pangolin lineage, and (ix) the date of release of sequences. In the second step, the number of protein sequences is shown. In the third step, the program will run the MSA using MAFFT (28), allowing the use of multiple cores to perform the alignment, optimizing the processing time. Then, a conservation score will be computed based on a scoring method and a BLOSUM matrix. We provide different options in regards to conservation scoring methods and give recommendations of which one to use inside the workflow. The final step of this workflow allows the user to compute and select peptides that belong to conserved regions of the protein alignment. Here SARS-Arena allows the selection of peptides with different lengths using the “min_len” and “max_len” variables. To guide the selection of peptides, we offer an interactive plot interface (**Figure 3**) where the user can set the conservation threshold, the rolling median window length, and the peptide length. The conservation threshold step, the selection of peptides, and the interactive plot interface are the same for Workflows 1A, 1B, and 1C; ergo they are described only in this subsection.

#### 3.1.2 Workflow 1B

Workflow 1B allows users to recover information from a pre-computed multiple sequence alignment. We recommend the use of this workflow for cases where there is a need to analyze a large number of protein sequences (e.g., more than 50,000 sequences). This workflow consists of three steps. In the first step, after importing the necessary libraries and setting a working directory, the user should set a month and a year to recover the pre-computed alignment. This option is given because there can be differences in the alignments obtained from N protein sequences released on different dates. This pre-computed alignment for each month/year combination is performed every week and stored at Owl Research Infrastructure Open Nebula (ORION) Virtual Machines Pool at Rice University. The Workflow 1B proceeds with the computation of the conservation score and the rest of the steps outlined in Workflow 1A.

#### 3.1.3 Workflow 1C

Workflow 1C allows the user to analyze the N protein sequences from SARS-related coronaviruses, not only SARS-CoV-2. In the first step, after the initial settings, the user is required to fetch a precomputed MSA alignment, similar to Workflow 1B. After the alignment, a consensus sequence will be printed on the screen and used as input for the next step. In the second step, a new MSA will be performed using as input the consensus sequence from SARS-CoV-2 N protein alignment and a set of predefined N protein sequences (64 in total) from SARS-related coronaviruses obtained from NCBI Protein databank. After the alignment completion, the workflow follows the same final steps of previous workflows, generating a peptide list that can be used in Workflow 2.

### 3.2 Workflow 2: Peptide-HLA Prediction for Conserved SARS-CoV-2 Peptides

Workflow 2 provides a way to model the three-dimensional structure of selected peptides in the context of different HLAs. In the first step the user should provide two files. The first one contains the list of peptides derived from Workflow 1; and the second one with the name of the HLAs written in the format “Gene*Allele group:HLA protein” (e.g., A*02:01 for HLA-A*02:01; C*11:07 for HLA-C*11:07). Since HLA binding depends to some extent on peptide sequence features (45), in the second step we use MHCflurry to perform initial filtering aiming to keep only good binders for further structural modeling. In this way, we avoid the unnecessary modeling of peptide-HLA that would probably not represent a good target for T-Cell Receptors (TCRs). We set the default cutoff to 500 nM, but this value can be modified according to user needs. In the third step, the three-dimensional HLA structure is created through homology modeling. As the HLA sequence is retrieved from EBI, any HLA can be modeled by our method. In the fourth step, the peptides selected from step 2 are modeled in the context of the chosen HLAs using a pHLA modeling tool called APE-Gen (38). We know that peptide-binding scoring functions are not completely accurate, but the use of multiple scoring functions can help to overcome this issue (46). For this reason, in the fifth and final step, we offer the opportunity to rescore the pHLAs generated by APE-Gen using different scoring functions. We added well-known scoring functions - Vina, Vinardo, and AD4 scoring - as well as a new machine learning-based scoring function recently developed, called 3pHLA (43).

### 3.3 Workflow Usage: A Case Study

A recent study revealed that HLA-B7^+^ individuals that recovered from COVID-19 disease triggered a cellular immune response against peptides from SARS-CoV-2 N protein (47). They identified one immunodominant epitope (SPRWYFYYL, hereafter referred to as SPR) that is conserved across different circulating coronaviruses. To assess if this epitope could be identified and selected by SARS-Arena, we start executing workflows 1A and 1C. The rationale was to generate two different lists of peptides, one from a direct comparison of N protein sequences from SARS-CoV-2 (Workflow 1A) and another one from the comparison of N protein sequences from SARS-related coronaviruses (Workflow 1C). For both workflows, we set an arbitrary conservation threshold of 85%. We were able to retrieve 41 and 17 peptide sequences from workflow 1A and1C, respectively. In the output, the SPR epitope was present in both lists (**Table 1**).

**Table 1:** Peptides above the conservation threshold of 85% selected from Workflow 1A and 1C.

Workflow 1A		Workflow 1C
PQCLPNNTA	DYKYWPQIA	PQNQRNAPR
PQGLPNNTA	EYKHWPQIA	QRRPQGLPN
`PQGLSNNTA	NYKHWPQIA	RRPQGLPNN
PQVLPNNTA	DYKHWPQVA	RPQGLPNNT
PQGVPNNTA	AYKHWPQIA	PQGLPNNTA
PQGLPNNTV	DYKDWPQIA	QGLPNNTAS
PEGLPNNTA	DYKRWPQIA	GLPNNTASW
QCLPNNTAS	HYKHWPQIA	**SPRWYFYYL**
QGVPNNTAS	DYKHWSQIA	TDYKHWPQI
QGLPNNTVS	DYKHWPQIA	DYKHWPQIA
QGLSNNTAS	YKHWPQIAR	YKHWPQIAQ
EGLPNNTAS	YKHWPQIAL	KHWPQIAQF
QVLPNNTAS	YKQWPQIAQ	DAYKTFPPT
QGLPNNTAS	YKHWPQVAQ	AYKTFPPTE
SPRWYFYYI	YKHWPQIAQ	YKTFPPTEP
**SPRWYFYYL**	YKLWPQIAQ	LPQRQKKQQ
SPRWFFYYL	YKRWPQIAQ	PQRQKKQQT
SPKWYFYYL	YKDWPQIAQ	
YYKHWPQIA	YKYWPQIAQ	
DYKLWPQIA	YKHWSQIAQ	
DYKQWPQIA		

**Figure 4:**
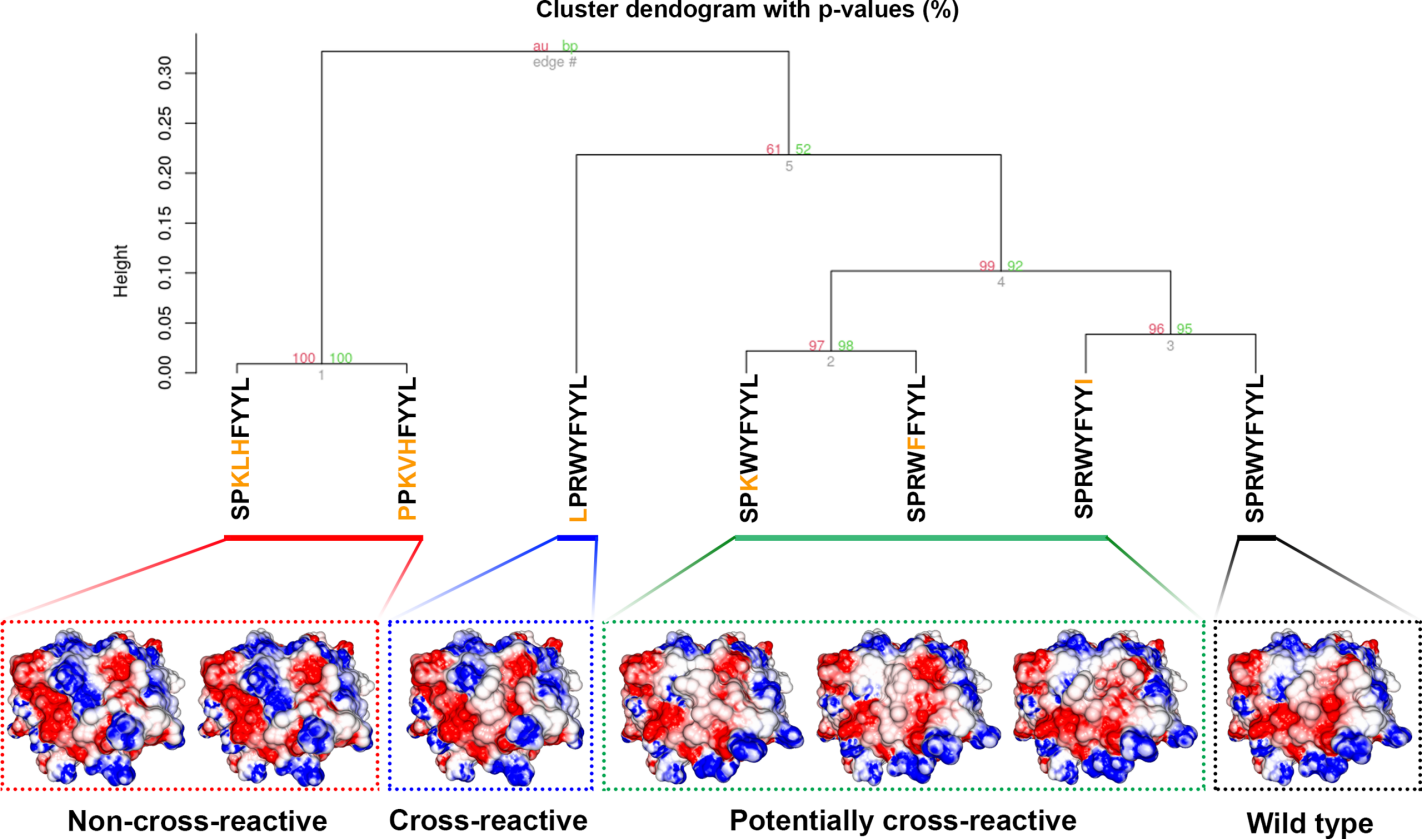
Hierarchical clustering analysis (HCA) and structure comparison of SPR peptide, SPR variants, LPR peptide, and SPK peptide. The TCR-interacting surface is shown in the pHLA complexes and the colors blue, white and red represent positive, neutral, and negative charges, respectively (+3kT to -3kT range). The SPR variants are closer to SPR peptide (wild-type), followed by the known cross-reactive LPR epitope. The non-cross-reactives epitopes SPK and PPK are grouped in a different branch of HCA, probably due to the central positive charge. Numbers in red and green represent the approximately unbiased p-value and the bootstrap probability value, respectively, for each cluster in the dendogram. Here we used the “correlation” as the distance measure and the “average” as the agglomerative method with a total of 100 bootstrap replications.

We also wanted to assess if the SPR epitope would be selected at the end of Workflow 2. Since this is an immunodominant epitope, we wanted to be sure SARS-Arena would filter this peptide out and rank it as one of the best peptide targets. For that, we used the peptide list from Workflow 1A and 1C as input to Workflow 2 along with a list of 10 prevalent HLAs (including the HLA-B*07:02 allele). Again, SARS-Arena identified the same epitope SPRWYFYYL described by Lineburg et al. (**Table 2**, in bold). Finally, one of the goals of SARS-Arena is also to identify peptide variants from conserved regions that can be used as possible targets in vaccine research. We noted similar sequences to SPRWYFYYL using the list of peptides from Workflow 1A (**Table 2**). The similarity of sequences, associated with the good HLA sequence-binding prediction and structural binding energy values of these epitopes, could indicate a possible cross-reactive response between these variants and the wild-type SPR epitope. To evaluate this possibility, we decided to use the three-dimensional models generated at the end of Workflow 2 to assess the probability of cross-reactivity based on electrostatic potential patterns from the pHLA surface, as previously described at (48, 49). We included in our analysis an SPR cross-reactive peptide (LPRWYFYYL, hereafter referred to as LPR) and two SPR non-cross-reactive peptides (PPKVHFYYL and SPKLHFYYL, hereafter referred to as PPK and SPK, respectively). Hierarchical clustering analysis revealed that the SPR epitope is more similar to the variants we have found than the known cross-reactive LPR peptide (**Figure 4**). Also, our analysis correctly separated non-cross-reactive epitopes PPK and SPK indifferent branches. The strong sequence and structure similarity set these variants as putative new targets to be tested towards the development of broad-spectrum T-cell vaccines.

**Table 2:** Identification of an immunodominant epitope in the context of HLA-B*07:02 and its variants. The lower the value the better.

Workflow	HLA Alelle	Peptide	Sequence-based prediction (nM)	Vina (kcal/mol)	Vinardo (kcal/mol)	AD4 score (kcal/mol)	p3HLA (nM)
**Workflow 1A**	B*07:02	**SPRWYFYYL**	11.37	-10.88	-15.11	-76.67	101.61
		SPRWFFYYL	14.89	-9.21	-12.34	-75.78	194.29
		SPRWYFYYI	25.11	-10.95	-14.90	-85.76	106.00
		SPKWYFYYL	68.47	-11.23	-15.69	-85.54	100.00
	B*40:01	YKHWPQIAL	181.24	-8.42	-11.42	-75.21	24428.89
**Workflow 1C**	B*07:02	**SPRWYFYYL**	11.37	-10.03	-14.42	-91.57	100.00
	A*24:02	KHWPQIAQF	258.11	-8.81	-12.33	-74.05	1112.68

*Immunodominant epitope is highlighted in bold.

## 4 Discussion

The SARS-CoV-2 pandemic highlighted the need for immunoinformatics approaches towards the identification of immunogenic protein targets. At first, the focus was on the humoral immune response. However, as it was recognized that cellular immunity plays an important role complementing or even filling the gap of humoral response, computational methods and databases focused on the prediction and analysis of SARS-CoV-2 T-cell epitopes have been developed (50–56). Here we presented SARS-Arena, a user-friendly environment for structure-guided epitope discovery targetting conserved regions of N protein from SARS-CoV-related viruses. SARS-Arena includes two customized workflows. Workflow 1 is focused on (i) fetching protein sequences from NCBI Virus and (ii) selecting of peptides found in conserved regions. Workflow 2 is focused on the three-dimensional modeling of peptides in the context of any HLA molecule. We run Workflow 1 and 2 to evaluate the capacity of SARS-Arena to identify and select epitopes in conserved regions. This analysis returned an immunogenic epitope (SPRWYFYYL) and possible cross-reactive variants. SARS-Arena goes beyond previous efforts by providing an easy-to-use environment for epitope discovery while integrating sequence and structure analysis and targetting conserved regions of SARS-CoV-related proteins.

The ultimate goal of SARS-Arena was to create a straightforward computational environment to enable epitope discovery efforts by basic, intermediate, and advanced users, while aggregating sequence- and structure-based analysis. Step-by-step workflows are provided as Jupyter Notebooks to be executed alongside tools provided in a Docker image, therefore facilitating the installation process. The workflows and supporting functions can also be modified by the user, to accommodate different computational and data analysis needs. Because SARS-Arena is highly modular and easy to customize, additional steps and functions for advanced practitioners can be implemented as needed.

We decided to focus SARS-Arena on the N protein because this protein is a promising target for broad-spectrum vaccine development. First, the N gene is more conserved and presents fewer mutations over time (57). Second, this protein is highly expressed upon infection, increasing the chances for epitopes to be presented to TCR scrutiny in the context of different HLAs (58). Previous studies have shown that N protein from SARS-CoV is highly immunogenic, and T-cell responses can persist for years after convalescence (59). Lastly, different regions of the N protein can pass through the intracellular antigen presentation pathway and be presented by a wide range of HLAs, eliciting a dominant cellular immune response (60). It is important to note that since we expect that some users may want to analyze other SARS-CoV-2 proteins, we also implemented Workflow 1A in a way that any SARS-CoV-2 protein can be analyzed. Finally, advanced users can also modify the provided functions and workflows to apply the same methods to proteins derived from other pathogens of interest.

Another novelty of SARS-Arena was the inclusion of 3pHLA, a scoring function that uses a per-peptide-position protocol to predict the binding affinity of pHLA complexes. Preliminary results show that 3pHLA outperforms widely used structural scoring functions (manuscript submitted). However, since the 3pHLA scoring relies on machine learning models trained on available binding affinity and structural data, this scoring function is currently restricted to a total of 28 HLA alleles.

We envision that SARS-Arena can be used as a tool to identify new targets to be used in broad-spectrum therapies. As a proof-of-concept, we decided to run SARS-Arena with a set of predefined parameters and compare the output with targets described in the literature. We focused our analysis on the SPRWYFYYL epitope. This epitope was involved in a dominant T-cell response in HLA-B7^+^ individuals, exposed or not to SARS-CoV-2 (47). In fact, this epitope has already been previously suggested as a SARS-CoV-2 target (61, 62). SARS-Arena not only was able to recover this peptide but also highlighted the presence of peptide variants in this region. We wonder if these variants could be cross-reactive targets. Surprisingly, in our analysis, the variants are closer to the wild-type peptide than the known cross-reactive target LPR. The analysis of pHLA surface in the context of electrostatic potential charges is robust, validated in previous studies, and has already been used to identify cross-reactive targets to an HCV peptide (63). Note that the pHLA structural modeling and analysis is crucial for this application since cross-reactivity can occur even among peptides with low sequence similarity and identity. Future studies will be required to fully validate novel targets identified with SARS-arena.

SARS-Arena can be used to identify and suggest new T-cell targets for SARS-CoV-2 and SARS-CoV-related protein sequences. This environment is simple, but still robust, offering end-to-end workflows to analyze these targets, from raw protein sequences to refined pHLA three-dimensional structures.

## Data Availability Statement

The original contributions presented in the study are included in the article/supplementary materials. Further inquiries can be directed to the corresponding authors.

## Author Contributions

MR, RF, AC, SH-S, DA, and LK contributed to conception and design of the study. MR wrote the manuscript, compiled the workflows, wrote the documentation, and set up the repository to storage of files. RF implemented the computer codes for Workflow 1, optimized the Workflow 2, and implemented the pre-computed alignments on ORION. AC implemented the computer codes of Workflow 2. LK and DA were responsible for the supervision, project administration, and funding acquisition. All authors contributed to manuscript revision, read, and approved the submitted version.

## Funding

This work was funded in part by the National Science Foundation IIBR: Informatics : RAPID program (2033262) and by Rice University funds. DAA and MMR are supported by a Computational Cancer Biology Training Program fellowship (CPRIT Grant No. RP170593). DAA is also supported in part by University of Houston funds. SHS is supported by a National Library of Medicine Training Program fellowship (T15LM007093-29). LEK is supported in part by NIH U01CA258512.

## Conflict of Interest

The authors declare that the research was conducted in the absence of any commercial or financial relationships that could be construed as a potential conflict of interest.

## Publisher’s Note

All claims expressed in this article are solely those of the authors and do not necessarily represent those of their affiliated organizations, or those of the publisher, the editors and the reviewers. Any product that may be evaluated in this article, or claim that may be made by its manufacturer, is not guaranteed or endorsed by the publisher.
